# Development of an attenuated sporozoite vaccine to prevent and eliminate *Plasmodium falciparum* malaria

**DOI:** 10.1186/1475-2875-9-S2-I1

**Published:** 2010-10-20

**Authors:** Peter F Billingsley

**Affiliations:** 1 Sanaria Inc., Rockville, Maryland, 20850, USA; 2 Naval Medical Research Center, US Military Malaria Vaccine Program, Silver Spring, Maryland 2091, USA; 3 Center for Vaccine Development, University of Maryland School of Medicine, Baltimore, Maryland 21201-1559, USA; 4 Radboud University Nijmegen Medical Center, Nijmegen,the Netherlands; 5 Leiden Malaria Research Group, LUMC, Leiden, the Netherlands; 6 Columbia University Medical Center, New York, NY, USA; 7 Protein Potential LLC, Rockville, Maryland, 20850, USA; 8 Vaccine Research Center, NIAID, NIH, Bethesda, Maryland, 20892-6612, USA

## 

An ideal vaccine for malaria would target all stages of the parasite life cycle, and thereby prevent infection, severe disease and transmission. However, most malariologists agree that if only one stage is to be targeted, it should be the pre-erythrocytic stage, because induction of highly protective immune responses against this stage will prevent blood stage infection and thus disease and parasite transmission.

Our consortium is working to develop such a vaccine. The first-generation vaccine is a metabolically active, non-replicating *Plasmodium falciparum* sporozoite (PfSPZ) vaccine that is attenuated by irradiation. The first major challenge was to manufacture adequate quantities of such a vaccine that met regulatory standards for initial clinical trials, and demonstrate that it was safe, well tolerated and immunogenic in humans. This has been accomplished. The second major challenge is to determine how to administer for the first time in humans an unprecedented, non-replicating and metabolically active (live) whole-organism vaccine formulation composed of PfSPZ that measure 0.5-1.0 μm x 7-10 μm, to induce > 85% protection.

Volunteers immunized by intradermal (ID) or subcutaneous (SC) routes were protected in the first clinical trial. However, the number of protected subjects was low, and despite a dose response immunogenicity was sub-optimal. Animal studies show that intravenous (IV) immunization induces much better immunity and protection than immunization by the SC or ID routes. These data have been used to inform the design of the next clinical trial. Parallel efforts are underway to optimize non-IV administration of PfSPZ, and the efficiency and scale-up of manufacture.

We are also determining whether genetic targeting techniques can be used to create a parasite clone that will produce attenuated PfSPZ that are not only more potent, but also as safe as radiation-attenuated PfSPZ. Such PfSPZ could be non-replicating (as are radiation-attenuated PfSPZ), replication deficient, or replication competent, but avirulent. Our goal is to develop, license and deploy a highly effective pre-erythrocytic stage PfSPZ vaccine that prevents blood stage infection, disease, and transmission. Such a vaccine could be used at the community level in Pf elimination campaigns, and at the individual level for prevention of Pf malaria in infants, young children, and pregnant women in endemic areas, as well as individuals of all ages who travel to malaria-endemic areas.

